# Postpartum Women's Childhood Trauma and Postpartum Depressive Symptoms: A Network Analysis

**DOI:** 10.1155/da/6982348

**Published:** 2025-10-30

**Authors:** Yanchi Wang, Wei Huang, Xujuan Xu, Jian Gu

**Affiliations:** ^1^Nursing Department, Affiliated Nantong Hospital of Shanghai University (The Sixth People's Hospital of Nantong), Nantong, Jiangsu, China; ^2^Medical School of Nantong University, Nantong, Jiangsu, China; ^3^Nursing Department, Affiliated Hospital of Nantong University, Nantong, Jiangsu, China; ^4^Department of Epidemiology and Health Statistics, School of Public Health, Nantong University, Nantong, Jiangsu, China

**Keywords:** childhood trauma, depressive symptoms, network analysis, postpartum women

## Abstract

**Background:**

There is growing recognition of the connection between childhood trauma and postpartum depressive symptoms. However, the specific patterns and complex relationships among them remain largely unclear. This study employs network analysis to dissect the intricate associations between postpartum women's childhood trauma and postpartum depressive symptoms, aiming to lay a foundation for targeted interventions.

**Methods:**

A total of 625 mothers who were undergoing the 42-day postpartum checkups participated in this research. Participants completed the Edinburgh Postnatal Depression Scale (EPDS) and the Childhood Trauma Questionnaire–Short Form (CTQ–SF). Using network analysis, we constructed a network model, calculated the expected influence (EI) and bridge EI (BEI) of nodes, and estimated the network's stability and accuracy.

**Results:**

The findings indicate that E8 (I have felt sad or miserable), C14 (Family said hurtful or insulting things to me), and C13 (Family looked out for each other) exhibited the greatest EI within the network. Meanwhile, E10 (The thought of harming myself has occurred to me), C14 (Family said hurtful or insulting things to me), and C3 (People in my family called me things like “stupid,” “lazy,” or “ugly”) emerged as the crucial bridge symptoms.

**Conclusions:**

This study uses network analysis to reveal the complex relationship between childhood trauma and postpartum depression (PPD). It highlights that hurtful childhood family remarks play a crucial role in this complex web and that early-life psychological traumas are reflected in postpartum self-harm tendencies. The findings enable healthcare providers to create targeted interventions.

## 1. Introduction

The postpartum period is a time of remarkable physical, hormonal, and psychological changes for women. Postpartum depression (PPD), a complex and incapacitating mood disorder, has drawn extensive research attention [1]. Defined as a mental disorder featuring depressive emotions post-childbirth, it extends beyond a mere mood dip, incorporating symptoms like persistent sadness, anxiety, irritability, disinterest in the infant, self-blame, guilt, sleep disturbances, appetite loss, and even suicidal ideation, all of which impede mothers' normal lives and role performance [2, 3]. Its significance is profound. For mothers, it impairs both physical and mental health, delaying recovery and degrading quality of life [4]. For infants, depressed mothers may struggle with feeding, caregiving, and emotional bonding, hindering the infant's development in terms of security, cognition, and emotion [3]. In families, a mother's depression can strain marital relations, disrupt communication, and undermine family harmony [5]. It is estimated that approximately 14% of new mothers experience PPD symptoms [6]. The prevalence was not uniform across countries, varying from 5.0% to 26.32%, and developing countries, particularly China, had a notable prevalence of PPD [6]. This prevalence not only highlights the magnitude of the problem but also emphasizes the urgent need for a deeper understanding of its underlying factors.

Childhood trauma, which encompasses a wide range of adverse experiences such as physical, sexual, and emotional abuse (EA), and neglect, has been consistently implicated in the development of mental health disorders throughout adulthood [7, 8]. It is well-proven that childhood trauma strongly correlates with adult mental health disorders, especially depression. The psychological imprints of early traumas impede the development of coping skills and emotion regulation, heightening vulnerability to depressive episodes later [9]. This link extends to PPD as well. Women with a childhood trauma history face a notably increased risk of PPD. Unresolved early emotional wounds often resurface during pregnancy and childbirth, interacting with hormonal and psychological shifts. Combined, these make it arduous for them to endure the postpartum phase without falling into depression [10, 11]. Yet, despite growing evidence of this connection, the precise mechanisms between childhood trauma and PPD remain unclear. Traditional correlational and mediation research methods fall short in deciphering the complex factors tying early-life traumas to PPD. To address this, more innovative research is urgently needed to understand the link and devise better preventive and therapeutic strategies, as understanding this connection is vital for combating PPD effectively.

Network analysis, rooted in the network theory of mental disorders [12, 13], proposes that mental disorders arise from the complex interplay of symptoms that are interconnected in a network-like structure. In this framework, symptoms are conceptualized as nodes, and the causal interactions between them are represented as connections between these nodes. This approach suggests that mental disorders can be understood as systems of mutually reinforcing symptoms rather than as the result of a single underlying cause [12]. The network approach to psychopathology has gained significant traction in recent years, offering a novel way to study and understand the dynamics of mental disorders. Previous studies have begun to explore the potential of network analysis in understanding mental health disorders. For instance, in the context of PTSD, McNally et al. [14] used network analysis to identify the key symptoms and pathways that contribute to the maintenance of the disorder. In the field of depression, Fried et al. [15] demonstrated how network analysis could reveal the central and bridge symptoms that are crucial for understanding the progression of the illness. Most existing research on PPD has concentrated on more immediate factors such as hormonal changes, breastfeeding difficulties, and social support deficits [6, 16]. While these factors are undoubtedly important, overlooking the potential long-term impact of childhood trauma represents a significant omission. Similarly, studies on childhood trauma have often examined its association with general mental health outcomes in adulthood, without zooming in on the specific context of the postpartum period. However, to date, few studies have specifically focused on the application of network analysis to unravel the relationship between childhood trauma and PPD [17].

This study aims to fill this critical knowledge gap by employing network analysis to a sample of postpartum women. By identifying the central and bridge nodes within this network, we anticipate being able to pinpoint the most influential factors in the development of PPD among women with a history of childhood trauma. This knowledge could then be translated into more targeted and effective prevention and intervention strategies, ultimately improving the mental health and well-being of postpartum women and their families.

## 2. Methods

### 2.1. Participants

From December 2023 to June 2024, this research was conducted at postpartum clinics within three exemplary tertiary-level hospitals in Nantong. The participants comprised mothers attending their routine 42-day postpartum check-ups. Trained medical researchers, thoroughly briefed on the study's particulars, were tasked with questionnaire administration. They ensured mothers were well-versed in the study details, obtained their voluntary consent, and guided them through questionnaire completion. Mothers were presented with an informed consent form, which they perused and signed voluntarily, signaling their agreement to take part. During data collection, strict measures were implemented to safeguard the mothers' privacy and comfort. The inclusion criteria were set as: (1) being 18 or older, (2) having a singleton pregnancy, and (3) possessing the capacity to independently comprehend, read, and respond to questionnaire items. The exclusion criteria were set as: (1) severe obstetric complications during birth for either mother or infant, (2) the mother having a serious underlying disease such as a malignant tumor, and (3) a preexisting severe mental disorder or significant communication difficulties. In total, 625 eligible mothers were incorporated into the study.

### 2.2. Measures

#### 2.2.1. Basic Information Questionnaire

The basic information questionnaire was designed by the research team, covering both basic demographic information and factors related to childbearing.

#### 2.2.2. The Edinburgh Postnatal Depression Scale (EPDS)

The EPDS was employed to assess postpartum depressive symptoms [18]. It consists of 10 items, each scored on a four-point Likert scale ranging from 0 to 3, where 0 indicates the least severe manifestation and 3 represents the most severe. Mothers were asked to rate their experiences over the past 7 days for each item. The total score was obtained by summing up the scores of all 10 items, with a higher overall score suggesting a greater likelihood of PPD [19]. In China, a score of ≥ 10 has been commonly used as the threshold for screening PPD [20].

#### 2.2.3. The Childhood Trauma Questionnaire–Short Form (CTQ–SF)

The CTQ–SF is a widely used retrospective screening tool to assess an individual's experience of childhood abuse and neglect [21]. It is important to note that within this scale, item 10, 16, and 22 do not belong to any of the five subscales (EA, physical abuse [PA], sexual abuse [SA], emotional neglect [EN], and physical neglect [PN]) and, thus, are not involved in the scoring process [22]. The remaining 25 items are grouped into these five subscales, which measure the history and severity of childhood trauma. Each of these 25 items is rated on a 5-point Likert scale (1 = never true to 5 = very often true) [23]. The total score, which is calculated by aggregating the scores of these five subscales, reflects the overall trauma level, with higher values indicating more severe experiences. The cutoff scores were established as follows: PA = 8, PN = 8, SA = 8, EA = 10, and EN = 15 [24]. These cutoff scores were used to ascertain whether a participant had been affected by the relevant types of abuse and neglect.

### 2.3. Ethics

Ethical approval for this study was granted by the Ethics Committee of the Affiliated Hospital of Nantong University, under Approval Number 2022-K150-01. At the commencement of the questionnaire administration, comprehensive details regarding the study's objectives were furnished to all participants. Their involvement in the survey was entirely discretionary, and respondents were given firm assurances concerning the safeguarding of their confidentiality, privacy, and anonymity. They were clearly informed that they could exit the study at any point without facing any negative repercussions. The research team committed to handling all collected data with the utmost confidentiality, ensuring its sole utilization for research purposes.

### 2.4. Statistical Analysis

#### 2.4.1. Network Construction and Visualization

A Gaussian graphical model illustrating the relationship between childhood trauma and postpartum depressive symptoms in women was constructed and visualized. This was achieved by applying the extended Bayesian information criterion (EBIC) in conjunction with the least absolute shrinkage and selection operator (LASSO). The implementation was carried out using the qgraph R-package within Rstudio, version 4.0.2. After taking other variables into account, partial correlation analysis based on Spearman's rank correlation coefficients was adopted to identify the direct correlations between each pair of variables. In this network, nodes were used to represent the 35 subsymptoms of both childhood trauma and PPD. The connections between these nodes were depicted by edges. Specifically, blue edges indicated positive associations, while red edges signified negative ones. The thickness of the edges corresponded to the strength of the correlation. The network layout was determined using a modified version of the Fruchterman–Reingold algorithm, which positions connected nodes close to each other to provide an informative visualization.

#### 2.4.2. Centrality Estimation

The prominence of each symptom was evaluated by computing three principal centrality measures, namely strength, betweenness, and closeness. However, as Owczarek et al. [25] proposed, expected influence (EI) outperforms traditional centrality metrics, especially in networks containing negative correlations. Hence, EI was chosen as the primary criterion for assessing the significance of nodes. To identify bridge symptoms within the network, the R-package networktools was utilized to calculate the bridge EI (BEI). A higher BEI score implies a greater probability of the influence of the current community extending to adjacent communities. Additionally, the R-package mgm was employed to determine the predictability of eachnode, which helps in evaluating how well a node is understood by its surrounding nodes and the overall controllability of the network model.

#### 2.4.3. Stability and Accuracy Estimation

The R-package bootnet was utilized in a three-stage process to evaluate network reliability and accuracy [17]. First, the precision of the edge weights was determined by establishing 95% confidence intervals through a nonparametric bootstrapping technique with 1000 bootstrap samples. Second, the consistency of centrality measures was evaluated by computing the correlation stability coefficient (CS–C) via a case-dropping bootstrapping approach. A CS–C threshold of 0.25 was set for network stability, with a value above 0.5 being more preferable. Finally, differences in network characteristics were examined, specifically focusing on whether there were significant variations in edge weights, EIs, or BEIs, through bootstrapped difference tests.

## 3. Results

### 3.1. Sample Characteristics

The mean age of the 625 participants was 30.6 ± 3.7 years, of whom 232 (37.1%) had a bachelor's degree or less, and 393 (62.9%) had a bachelor's degree or above. A total of 388 (62.1%) lived in urban areas, 126 (20.1%) in towns, and 111 (17.8%) in rural areas. The prevalence of depression was 11.8% (*n* = 74), and 31.0% (*n* = 194) had experienced at least one childhood trauma.  Table 1 lists the abbreviations, average scores, and predictability for each node.

### 3.2. Network Structure

**Figure 1:**
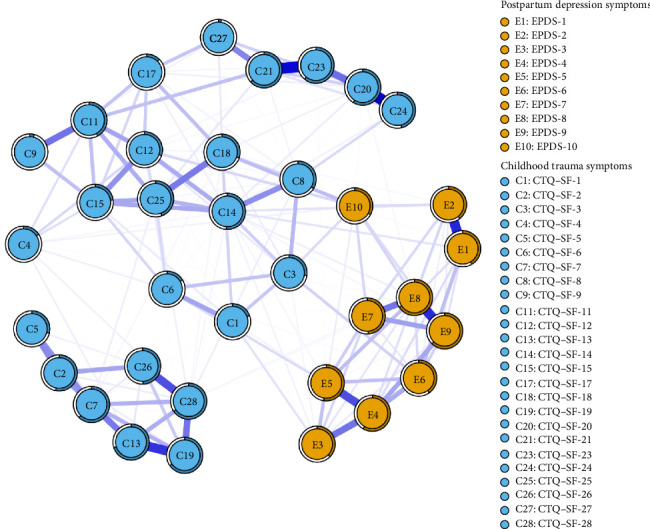
Network structure of maternal childhood trauma and postpartum depression symptoms.

Figure 1 illustrates the network structure involving childhood trauma and postpartum depressive symptoms, which consists of 35 nodes. The mean weight of the edges in this network is 0.080. In terms of the thickness of the edges, the connection between C20 and C24 stood out, with an edge weight of 0.452, making it the thickest. The second was the edge linking C21 and C23, which boasted an edge weight of 0.428. Additionally, the edge between E1 and E2 had an edge weight of 0.390, and the one connecting E8 and E9 had a weight of 0.389.

### 3.3. Node Centrality and Stability

**Figure 2:**
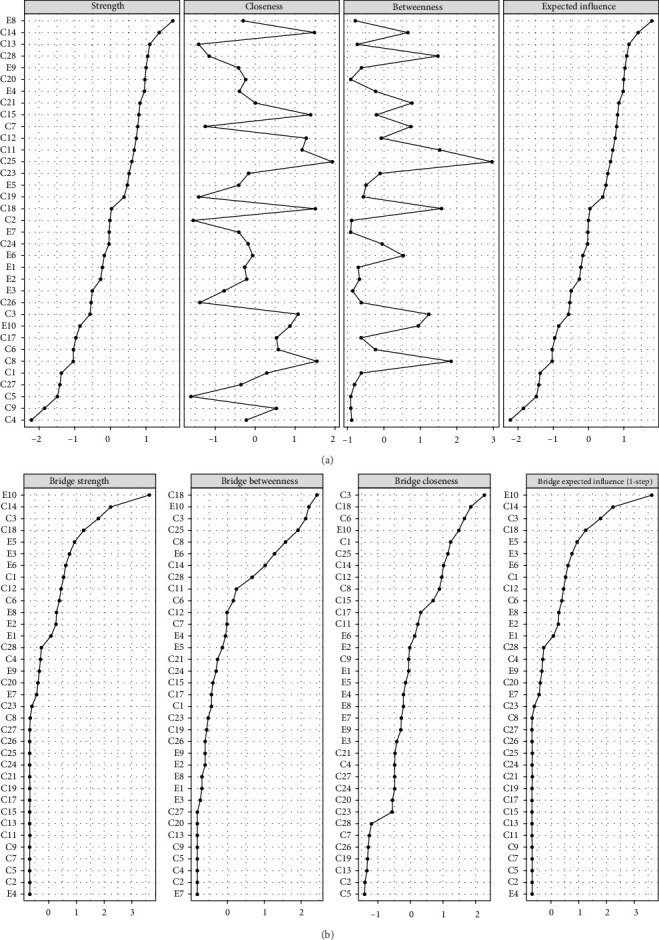
(A) EI of the network of maternal childhood trauma and postpartum depression symptoms. (B) BEI of the network of maternal childhood trauma and postpartum depression symptoms.

**Figure 3:**
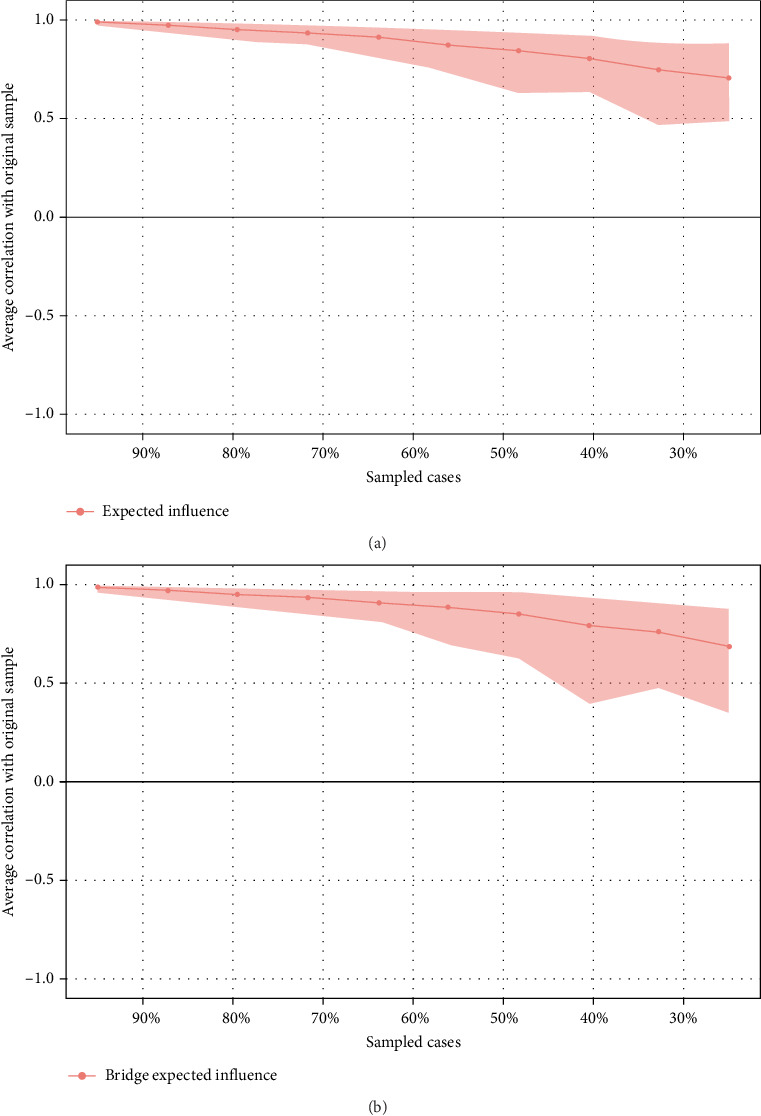
(A) Stability of expected influence indices using case-dropping bootstrap. (B) Stability of bridge expected influence indices using case-dropping bootstrap.

In Figure 2A, E8 (I have felt sad or miserable) has the highest EI at 1.186, followed by C14 (Family said hurtful or insulting things to me) with an EI of 1.103, and C13 (Family looked out for each other) at 1.044, marking them as the most central symptoms. Meanwhile, C4 (Parents too drunk or high to take care of me) with an EI of 0.310 and C9 (Hit hard enough to see doctor) with an EI of 0.391 have the lowest EIs, implying they may be on the periphery of symptomaticity in the network. The case-dropping bootstrapping results indicated a CS–C value of 0.517 for EIs. This means that when 70% of the original data is discarded, the centrality of the remaining 30% still correlates at 0.517 with that of the complete original dataset, demonstrating sufficient stability in the node EI estimates, as depicted in Figure 3A.

### 3.4. Bridge Nodes Centrality and Stability

E10 (The thought of harming myself has occurred to me) (BEI = 0.297) had the highest BEI, followed by C14 (Family said hurtful or insulting things to me) (BEI = 0.201) and C3 (People in my family called me things like “stupid,” “lazy,” or “ugly”) (BEI = 0.170). This implies they could be pivotal bridge contributing to childhood trauma and PPD symptoms. E4 (I have been anxious or worried for no good reason) had the lowest BEI at 0.027, suggesting it was the least likely to spread across different communities (Figure 2B). The case-dropping bootstrapping results show a CS–C value of 0.517 for BEIs, meaning there is a 0.517 correlation between the bridge centrality of the remaining 30% data and that of the full original dataset after eliminating 70% of the original data, signifying the node BEIs possess a satisfactory degree of stability, as illustrated in Figure 3B.

### 3.5. Node Predictability

As shown in Figure 1, the predictability of a node is manifested by the proportion of coloring on the surrounding circle. The predictability values of the nodes varied from 0.024 to 0.701, and the average predictability reached 0.416. This implies that, on average, the adjacent nodes contributed 0.416 to the variance of each individual node. Among them, node C13 exhibited the highest predictability at 0.701, with E8 following closely at 0.658. In contrast, C27 had the lowest predictability, merely 0.024.

## 4. Discussion

This study utilized the psychological network analysis method to explore the complex interactions between maternal childhood trauma and postpartum depressive symptoms. Three core symptoms were identified, namely E8 (I have felt sad or miserable), C14 (Family said hurtful or insulting things to me), and C13 (Family looked out for each other), which might be the keys to triggering and maintaining the network of childhood trauma and postpartum depressive symptoms. Finally, we determined that E10 (The thought of harming myself has occurred to me), C14 (Family said hurtful or insulting things to me), and C3 (People in my family called me things like “stupid,” “lazy,” or “ugly”) were the bridge symptoms that increased the risk and severity of childhood trauma and postpartum depressive symptoms. Apparently, the hurtful and insulting words from family members play a significant role in the symptom network of childhood trauma and PPD, acting as triggers and linkages that set off and interconnect the entire network.

Prior research has indicated that in networks comprising various communities, the most robust connections typically occur within communities rather than between them [27]. The network analysis conducted in this study corroborated this observation. In particular, the most intense links were observed within the PPD community (e.g., E1–E2 and E8–E9) and the childhood trauma community (e.g., D20–D24 and D21–D23). The strong association between E1–E2 and E8–E9 is supported by previous studies [28]. Previous studies on childhood trauma communities are mostly based on five dimensions, and this study can be more detailed to each item itself according to study [26, 29].

In our study, item E8 (I have felt sad or miserable) of the EPDS emerges as the most crucial symptom within the network interconnecting childhood trauma and PPD. It serves as a significant manifestation of the link between the two. Childhood trauma, as established by prior research, predisposes women to a heightened risk of PPD [30]. The prominence of E8 may be attributed to the fact that the feelings of sadness it represents are often rekindled or exacerbated during the postpartum period due to the stressors associated with childbirth and new motherhood, especially for those with a history of childhood trauma [31]. This symptom could act as a conduit, channeling the latent psychological distress seeded by childhood adversities into the realm of postpartum depressive symptoms [32]. This symptom's centrality within the network is further evidenced by its far-reaching connections. Understanding E8 as this crucial nexus allows healthcare providers to adopt a more refined approach during postpartum assessments. Instead of a broad-brush examination of depressive symptoms, they can zero in on E8. By recognizing it as the key indicator, they can meticulously screen for the underlying childhood trauma history and ultimately engineer more precisely targeted interventions. These interventions could span from evidence-based trauma therapies that help mothers process and heal their past traumas to practical support strategies like providing respite care to ease the burden of new motherhood. In this way, by leveraging the understanding of E8's role, healthcare providers can offer mothers in this vulnerable period a more robust shield against the encroachment of depression, safeguarding their mental well-being and facilitating a smoother transition into motherhood.

This study firmly establishes that item E10 (The thought of harming myself has occurred to me) of the EPDS represents the most critical bridging symptom. The significance of this bridging role is far-reaching. As a central symptom node, it wields predictive power over other nodes within the symptom network. Consequently, when healthcare providers conduct postpartum evaluations of new mothers, they must give heightened attention to E10. Through meticulous and continuous monitoring of any changes in E10, they can gain early indications of potential mental health issues. Once E10 shows signs of deterioration, it should promptly trigger a comprehensive investigation into the woman's childhood trauma history. This should involve a detailed exploration of the nature, frequency, and severity of the traumas she experienced during childhood. Simultaneously, a thorough assessment of current stressors such as breastfeeding difficulties, relationship tensions with partners or family members, and financial hardships is essential. It is important to note that existing research has definitively demonstrated a strong connection between E10 and postpartum suicidal behaviors [33]. Additionally, studies have suggested that emotional dysregulation stemming from childhood trauma may underlie self-harm tendencies [34, 35]. Hence, trauma-informed cognitive–behavioral therapy should be carefully customized to the woman's specific trauma history. This may incorporate techniques like exposure therapy to help desensitize her to trauma-related triggers, cognitive restructuring to modify negative self-perceptions and beliefs formed as a result of the trauma [36], and emotion regulation training to enhance her ability to manage intense emotions. By implementing such a comprehensive and targeted approach, the progression of symptoms can be effectively interrupted, and the woman's mental well-being safeguarded.

From a developmental psychology perspective, childhood is a crucial stage for personality development and self-awareness formation. During this period, the family environment, especially the verbal communication patterns among family members, wields profound influence. Verbal abuse, such as family members using hurtful and insulting language, can act as a “core catalyst” in the development of mental health issues later in life [37]. Research by Tebeka et al. [38] has established a significant link between childhood trauma, particularly EA involving verbal attacks, and an increased risk of depression in adulthood, notably during the perinatal period. Their findings indicate that women with a history of such childhood trauma are more predisposed to PPD. This is further supported by neurobiological research [37], which suggests that specific brain regions and pathways involved in processing aversive experiences are targeted by parental verbal abuse. This could potentially lay the groundwork for vulnerability to PPD. Landman et al. [39] explored the relationship between postpartum blues and PPD, highlighting various factors like personal psychiatric history, obstetrical elements, and stressful life events. Although not directly focused on family verbal abuse, it is reasonable to infer that this form of abuse can interact with these factors. For instance, women who endured childhood EA might be more sensitive to stressful life events during pregnancy. Their ability to cope with the challenges of childbirth and the postpartum period is likely diminished, thus heightening their susceptibility to PPD [40]. On a social level, it becomes evident that constructing a healthy family verbal environment is of utmost urgency. Family therapy interventions can play a pivotal role in making family members aware of the power of their words and guiding them to modify communication patterns. Coupled with providing early psychological counseling for expectant mothers with a history of childhood trauma, this dual approach has the potential to break the “chain reaction” that frequently leads from childhood trauma, exacerbated by verbal abuse, to PPD. Not only does this safeguard the mental health of mothers and their children, but it also promotes healthier family relationships and contributes to a more harmonious social atmosphere overall. By addressing the root cause of the problem—the damaging effects of verbal abuse within the family—we can take a significant step toward preventing the onset of PPD and fostering the well-being of families.

This study has some limitations. First, the sample was recruited from only three tertiary hospitals in Nantong, which might limit the generalizability of our findings. There could be regional differences in postpartum women's experiences and mental health status that were not captured, as different cultural, economic, and social backgrounds across various regions might influence the prevalence and manifestations of childhood trauma and postpartum depressive symptoms. Second, the cross-sectional nature of this study precludes the establishment of causal relationships. Longitudinal studies are warranted to track the development of these symptoms over time and clarify the causal directions. Finally, the study did not account for potential confounding factors comprehensively. Other variables such as recent life stressors, social support systems beyond the family, and preexisting mental health conditions prior to pregnancy might interact with childhood trauma and influence postpartum depressive symptoms. Future studies should integrate a more extensive array of factors to bolster the models' explanatory capacity.

## 5. Conclusions

This study has significantly advanced our understanding of the relationship between postpartum women's childhood trauma and postpartum depressive symptoms. This discovery offers crucial insights for understanding the underlying mechanisms and provides a foundation for devising effective preventive and therapeutic strategies. The key nodes and bridge symptoms within the network of postpartum women's childhood trauma and postpartum depressive symptoms have been successfully identified. This discovery offers significant insights into understanding the relationship network between maternal childhood trauma and PPD and lays a solid foundation for formulating effective preventive and treatment strategies. These findings serve as a cornerstone for healthcare providers to formulate targeted interventions, thereby enhancing the mental well-being of postpartum women and promoting the healthy development of both mothers and infants.

## Figures and Tables

**Table 1:** Descriptive statistics of the items in the postpartum women's childhood trauma and postpartum depressive symptoms network.

Abbreviation	Symptoms	Mean ± SD	Predictability
EPDS-1	I have been able to laugh and see the funny side of things	1.17 ± 0.42	0.414
EPDS-2	I have looked forward with enjoyment to things	1.13 ± 0.38	0.392
EPDS-3	I have blamed myself unnecessarily when things went wrong	1.66 ± 0.80	0.374
EPDS-4	I have been anxious or worried for no good reason	1.74 ± 0.79	0.586
EPDS-5	I have felt scared or panicky for no very good reason	1.39 ± 0.64	0.523
EPDS-6	Things have been getting on top of me	1.50 ± 0.67	0.402
EPDS-7	I have been so unhappy that I have had difficulty sleeping	1.33 ± 0.62	0.491
EPDS-8	I have felt sad or miserable	1.41 ± 0.67	0.658
EPDS-9	I have been so unhappy that I have been crying	1.46 ± 0.70	0.624
EPDS-10	The thought of harming myself has occurred to me	1.06 ± 0.30	0.332
CTQ–SF-1	Not enough to eat	1.09 ± 0.38	0.197
CTQ–SF-2	Someone to take care of and protect me	2.18 ± 1.54	0.532
CTQ–SF-3	People in my family called me things like “stupid,” “lazy,” or “ugly”	1.26 ± 0.60	0.276
CTQ–SF-4	Parents too drunk or high to take care of me	1.09 ± 0.46	0.157
CTQ–SF-5	Made to feel important	3.33 ± 1.61	0.289
CTQ–SF-6	Wore dirty clothes	1.16 ± 0.51	0.164
CTQ–SF-7	Felt loved	1.97 ± 1.43	0.622
CTQ–SF-8	Parents wished I had never been born	1.11 ± 0.39	0.335
CTQ–SF-9	Hit hard enough to see doctor	1.02 ± 0.20	0.040
CTQ–SF-11	Hit hard enough to leave bruises	1.03 ± 0.18	0.420
CTQ–SF-12	Punished with hard objects	1.08 ± 0.31	0.303
CTQ–SF-13	Family looked out for each other	1.99 ± 1.40	0.701
CTQ–SF-14	Family said hurtful or insulting things to me	1.18 ± 0.54	0.509
CTQ–SF-15	Physically abused	1.05 ± 0.25	0.227
CTQ–SF-17	Got hit badly enough to be noticed by teacher, neighbor, or doctor	1.02 ± 0.21	0.125
CTQ–SF-18	Felt hated by family	1.03 ± 0.20	0.303
CTQ–SF-19	Family felt close	1.94 ± 1.30	0.654
CTQ–SF-20	Someone tried to touch me in a sexual way or tried to make me touch them	1.06 ± 0.26	0.589
CTQ–SF-21	Hurt me unless I did something sexual with them	1.03 ± 0.23	0.633
CTQ–SF-23	Made to do sexual things	1.05 ± 027	0.610
CTQ–SF-24	Someone molested me	1.05 ± 0.25	0.463
CTQ–SF-25	I was emotionally abused	1.05 ± 0.28	0.444
CTQ–SF-26	Someone to take me to the doctor if I needed it	1.95 ± 1.49	0.496
CTQ–SF-27	I was sexually abused	1.01 ± 0.11	0.024
CTQ–SF-28	My family gave strength and support	1.79 ± 1.29	0.657

## Data Availability

The data that support the findings of this study are available on request from the corresponding author. The data are not publicly available due to privacy or ethical restrictions.
